# Tldi1 deficiency mediates Tlde1 self-toxic effect to regulate the adaptability of *Salmonella* Typhimurium infection and host immune response in mice

**DOI:** 10.3389/fcimb.2026.1902089

**Published:** 2026-08-03

**Authors:** Junfeng Zhang, Chang Liu, Songbiao Chen, Rongxian Guo, Yanyan Jia, Jing Li, Chengshui Liao, Ke Ding, Lei He, Ke Shang

**Affiliations:** 1 College of Medical Technology and Engineering, Henan University of Science and Technology, Luoyang, China; 2 College of Animal Science and Technology/Laboratory of Functional Microbiology and Animal Health, Henan University of Science and Technology;, Luoyang, China; 3 College of Animal Science and Technology, Key Laboratory of Live Carrier Biomaterials and Animal Disease Prevention and Control, Luoyang, China; 4 College of Animal Science and Technology, Henan Institute of Science and Technology, Xinxiang, China

**Keywords:** environmental stress adaptation, host-pathogen interactions, *Salmonella* Typhimurium, T6SS, Tldi1

## Abstract

**Introduction:**

Type VI secretion system (T6SS) is a key bacterial secretion device in *Salmonella enterica* serovar Typhimurium (*S*. Typhimurium), responsible for translocating effectors to mediate bacterial-host interaction. Tldi1, a well-characterized immunity protein encoded in the T6SS gene cluster, specifically neutralizes the cognate toxin Tlde1 to prevent bacterial “friendly fire”. However, there is still a lack of systematic and in-depth research on the functions of Tldi1 at present. In order to elucidate the phenotype and toxicity effects of Tldi1 deficiency on *S*. Typhimurium, this study used *S*. Typhimurium SL1344 as a model strain and explored the role of Tldi1 toxicity on bacterial ecological adaptability and infection process through multi-level experiments.

**Methods:**

Firstly, molecular bioinformatics approaches were employed to analyze the physicochemical properties, hydrophilicity, hydrophobicity, transmembrane regions, subcellular localization, as well as secondary and tertiary structures of the Tldi1 protein. Secondly, a *tldi1*-deficient mutant of *S*. Typhimurium was constructed via homologous double crossover recombination, and alterations in its biological characteristics were analyzed. Finally, a mouse infection model was used to investigate the effect of *tldi1* deletion on the infection progression of *S*. Typhimurium.

**Results:**

The results showed that compared with the wild-type (WT) strain, the Δ*tldi1* strain exhibited reduced splenic colonization, and regulated host inflammation, while these phenotypes were reversed in the Δ*tldi1/p*Δ*tldi1* strain.

**Discussion:**

Tldi1, as a key immunity protein for Tlde1 neutralization, indirectly regulates the environmental adaptability and host immune homeostasis of *S.* Typhimurium, providing new insights into the function of T6SS immunity proteins.

## 1 Introduction

*Salmonella* represents a major group of Gram-negative, facultatively anaerobic bacilli and is a widespread zoonotic pathogen capable of causing various intestinal diseases, thereby posing significant threats to both human and animal health (Fröder et al., 2026). To date, over 2,600 serotypes of *Salmonella* have been identified, with notable examples including *Salmonella enterica* serovar Typhimurium (*S*. Typhimurium), *S*. Enteritidis, and *S*. Choleraesuis. Among these, *S*. Typhimurium stands out as one of the primary serotypes responsible for foodborne outbreaks and epidemics (Zheng et al., 2025). The pathogenicity of *Salmonella* relies on multiple virulence determinants, including the type III secretory system (T3SS) (Gül et al., 2024) and effector proteins encoded by SPI-1 (*Salmonella* pathogenicity island, SPI) and SPI-2 (Ge et al., 2025), adhesion factors, lipopolysaccharide (LPS) and its modification system, antioxidant/stress response factors, metal acquisition systems, and regulatory networks. Emerging evidence indicates that the T6SS further contributes to *Salmonella*’s virulence mechanisms (Blondel et al., 2023; Troxell, 2018).

The T6SS is a widely distributed protein delivery apparatus in Gram-negative bacteria that directly translocates effector proteins into neighboring bacterial or eukaryotic host cells (Sana et al., 2016). Structurally, it resembles an inverted bacteriophage tail apparatus (Kielkopf et al., 2024). The T6SS consists of 13 core structural components (TssA-TssM, ClpV, Hcp, and VgrG) that form its functional framework, along with numerous effector proteins that determine its biological specificity. These effectors serve as the system’s “molecular ammunition”, with each possessing distinct functional properties (Ahmad et al., 2020; Mulder et al., 2012). Growing evidence demonstrates that *Salmonella* leverages its T6SS to secrete toxic effectors into competing bacteria, eliminating microbial rivals to secure ecological advantages (Blondel et al., 2023; Jurėnas et al., 2022). Notably, Hcp, functioning as both a structural component and effector, facilitates *Salmonella* invasion, adhesion, and virulence augmentation (Song et al., 2022). The T6SS(SPI-6) and T6SS(SPI-19) systems in *Salmonella* Dublin CT_02021853 mediate interbacterial competition (Amaya et al., 2022). The T6SS-ClpV complex enhances *S*. Typhimurium’s environmental adaptability, promoting intestinal adhesion, colonization, and microbiota dysregulation (Chen et al., 2025, 2024).

The T6SS as a key contact dependent secretion device for Gram negative bacteria, relies on the classical toxin antitoxin (TA) module composed of effector immunoglobulin pairs. Effect proteins exert toxic effects through enzymatic activities such as nucleases and phospholipases, mediating bacterial competition or host interactions; Immunoprotein specific binding effector proteins neutralize their toxicity and prevent self-toxicity of bacteria and homologous strains (Le et al., 2023; Zhang et al., 2013). At present, research has mainly focused on the toxic mechanisms of effector proteins and the neutralizing effects of immune proteins. However, there is still a lack of systematic analysis on the survival strategies of bacteria in complex microbial environments, their interaction patterns with competing strains, and their impact on host immune responses after the loss of immune protein genes. Domains of Unknown Function (DUFs) represent a class of conserved protein domains characterized by two key features: evolutionarily preserved amino acid sequences and undefined biological roles (Lv et al., 2023). Sibinelli-Sousa S et al. identified an immune protein containing the DUF2195 domain and named it Tldi1, this protein acts as an antitoxin that neutralizes the toxic activity of the effector protein Tlde1 (Lorente Cobo et al., 2022; Sibinelli-Sousa et al., 2020). Although Tldi1 has excellent characteristics as an immune protein of Tlde1, and most studies have focused on its role *in vitro* bacterial competition, its specific biological functions and impact on gut microbiota homeostasis during *S*. Typhimurium infection have not been fully studied in terms of its mechanism of action. This study constructed a *tldi1* gene deficient strain using homologous recombination double exchange technique, and compared and analyzed the differences in growth characteristics, bacterial competitiveness, and host gut colonization ability between mutant and WT strains. This provides experimental evidence for elucidating the regulatory role of T6SS immune protein Tlde1 in the interaction between *S*. Typhimurium and host gut microbiota after its deletion.

## 2 Materials and methods

### 2.1 Animals and ethical statement

Specific pathogen-free, 4-week-old Kunming (KM) female mice were purchased from Henan Scribes Biotechnology Co., Ltd. All animal experimental procedures were approved by the Institutional Animal Care and Use Committee (IACUC) of the Laboratory Animal Center, Henan University of Science and Technology, and all experiments were performed in strict accordance with the institutional ethical guidelines (Approval No. HAUST-025-M0317025).

### 2.2 Plasmid vectors and strains

The WT strain *S*. Typhimurium SL1344 (GenBank: FQ312003.1) served as the parental strain for constructing the *tldi1* gene deletion mutant (Δ*tldi1*). Primer sequences used for mutant generation are provided in **Supplementary Table 1**, and plasmid details are summarized in **Supplementary Table 2**. All bacterial strains were routinely cultured on Luria−Bertani (LB) solid and liquid medium with appropriate antibiotic supplementation: 25 μg/mL Chloramphenicol or 100 μg/mL Ampicillin. Plasmids pDM4 and pBR322 were maintained under standard laboratory conditions throughout the study.

### 2.3 Bioinformatics analysis

Bioinformatics analysis was performed using multiple computational tools (**Table S3**) to examine the amino acid sequence and predict the structural characteristics of the Tldi1 protein.

### 2.4 Construction of *S*. TyphimuriumΔ*tldi1* deletion and complementation strains

Using *S*. Typhimurium SL1344 genomic DNA as a template, the upstream (*tldi1*-Up) and downstream (*tldi1*-Down) homologous arms of the target gene were amplified separately using the primer pairs *tldi1*-Up-F1/R1 and *tldi1*-Down-F2/R2. These homologous arms were subsequently joined by overlap extension PCR using external primers *tldi1*-F1/R2 to generate the fused fragment *tldi1*-Up-Down. The pDM4 vector was then linearized by *Xho* I restriction enzyme digestion and ligated with the *tldi1*-Up-Down fragment using seamless cloning enzymes. The ligation products were transformed into *E. coli* SM10 competent cells via heat shock transformation (42°C, 45 s). Positive transformants were screened through *Xho* I restriction digestion and verified by colony PCR amplification with primers *tldi1*-F1/R2, ultimately yielding the confirmed recombinant plasmid pDM4-Δ*tldi1*. Extract plasmids using plasmid extraction kit and sequence them using Sanger sequencing to confirm the correct insertion of pDM4-Δ*tldi1.*

Conjugation transfer was carried out between donor strain *E. coli* SM10 harboring pDM4-Δ*tldi1* and recipient strain *S*. Typhimurium SL1344. Following successful conjugation, a single positive colony was selected and verified by PCR amplification using primers *tldi1*-F1/R2. The confirmed transconjugant was cultured in LB broth supplemented with chloramphenicol (Cm) at 37°C, then sub-cultured at 1:100 dilution in NaCl-free LB medium (NB medium) containing 20% sucrose for counter-selection. Subsequent streaking was performed on tri-sector LB agar plates supplemented with 20% sucrose (NaCl-free) to isolate individual clones. Candidate colonies were screened through PCR using both target-specific primers (*tldi1*-F1/R2) and chromosomal control primers (invA-F/R). Finally, the deletion strain identified by PCR were sent to genewiz Biotechnology Co., Ltd. for Sanger sequencing, and it was confirmed that the construction of the missing strains was successful.

The target gene was amplified by PCR using primers *tldi1*-H-F/R (**Table S1**), followed by purification of the PCR product with a DNA gel recovery kit (E.Z.N.A.^®^ Gel Extraction Kit). Concurrently, the pBR322 plasmid was linearized through single digestion with *Sal* I. Subsequently, the target gene fragment and linearized plasmid were ligated using a one-step cloning kit (10923ES-Hieff Clone^®^ Universal II One Step Cloning Kit) and transformed into *E. coli* DH5α competent cells through heat shock. Positive transformants were screened by colony PCR amplification using primer *tldi1*-F/R to confirm successful plasmid construction. The recombinant plasmids were extracted using a plasmid extraction kit and sent to Genewiz Biotechnology for Sanger sequencing analysis. After successful sequencing, the recombinant plasmids were extracted again. Ten microliters of plasmid DNA were transformed into the *S.* Typhimurium *tldi1* deletion strain using a SCIENTZ-2CS gene pulser with the following parameters: 2 kV, 200 Ω, and 25 μF.

### 2.5 Bacterial growth characteristics and environmental stress resistance assessment

The growth characteristics of WT, Δ*tldi1* and Δ*tldi1/p*Δ*tldi1* strains were evaluated in both standard LB medium and LB supplemented with 0.05% bile salts by measuring OD_600_ values at hourly intervals to generate growth curves. Parallel environmental stress tolerance assays were conducted wherein each strain was inoculated into modified LB media containing: (1) varying pH levels, (2) different NaCl concentrations (0–5%), (3) graded ethanol concentrations (0–5% v/v), and (4) incremental bile salt concentrations (0–0.25%). All cultures were placed in 250 mL conical flasks and shaken for 6 h at 37 °C and 180 r/min, after which OD_600_ was measured to quantitatively assess stress resistance profiles.

### 2.6 Bacterial biofilm formation assay

Biofilm formation was assessed with slight modifications to established protocols (Wang et al., 2025a, 2025). Overnight cultures of the experimental strains were standardized to OD_600_ = 0.1 and diluted it in LB medium at a ratio of 1:100. Subsequently, 100 μL of each diluted bacterial suspension was aliquoted into the wells of a 96-well plate and incubated at 37°C for 24 h or 48 h. After incubation, planktonic cells were removed, was gently washed the adhered biofilm three times with PBS, fix with methanol, and air dry. The biofilms were then stained with crystal violet, washed again three times with PBS, and destained with 95% ethanol to solubilize the retained dye. The absorbance at 595 nm (OD_595_) was measured using an automated microplate reader for quantitative biofilm assessment. All experiments had 6 biological replicates each time, and the experiments were repeated three times. Data are presented as the average. For biofilm composition analysis, bacterial strains were cultured on LB solid agar supplemented with either Congo red (40 μg/mL) or Coomassie brilliant blue (20  μg/mL), and colony morphology and pigmentation were examined after incubation (Gu et al., 2022).

### 2.7 Bacterial motility assay

The motility assay was performed following previously described methods with minor modifications (Williams et al., 2025). Swimming motility was assessed by inoculating WT, Δ*tldi1* and Δ*tldi1/p*Δ*tldi1* strains onto 0.3% semi-solid LB agar plates, followed by static incubation at 37°C for 6 h, after which motility zones were examined and their diameters measured for quantitative comparison.

### 2.8 Animal experiments

#### 2.8.1 Mouse virulence assay

Seventy-eight healthy KM mice with matched body weights were randomly allocated into 13 groups (6 mice per group), including six experimental groups each for WT and Δ*tldi1* infection and one PBS control group. Bacterial suspensions (0.2 mL/animal) ranging from 1.0×10^9^ to 1.0×10^4^ CFU/mL were administered via intraperitoneal injection, while control mice received equivalent volumes of sterile PBS. Mortality was recorded at 12 h intervals for 21 consecutive days, after which the 50% lethal dose (LD_50_) was calculated using Kou’s modified Karber method (Song et al., 2020).

#### 2.8.2 Mouse virulence assay Bacterial load, body weight changes, and organ indices

Twenty-four weight-matched healthy Kunming mice were randomly divided into four experimental groups, with six animals per group. Each group received intraperitoneal injections (0.2 mL/mouse) containing 1.0×10^7^ CFU/mL suspensions of either WT, Δ*tldi1*, or Δ*tldi1/p*Δ*tldi1* strains. At 3 dpi and 5 dpi, three mice per group were humanely euthanized by cervical dislocation, followed by aseptic removal of livers and spleens. Organ weights were recorded and normalized to body weight (organ index=organ weight/body weight). For bacterial enumeration, tissues were homogenized in PBS (900 μL per 0.1 g tissue), serially diluted ten-fold, plated for viable counts, and repeated in triplicate to determine tissue bacterial loads.

#### 2.8.3 Pathological analysis of various organ tissues

Liver, spleen, and cecum tissues from each experimental group were aseptically collected at the designated timepoint (Section 2.7.2) and subsequently submitted to Wuhan Servicebio Co., Ltd. for comprehensive histopathological evaluation, with cecal specimens undergoing additional Alcian Blue-Periodic Acid Schiff (AB-PAS) staining analysis.

#### 2.8.4 RNA extraction and real-time quantitative PCR determination

The total RNA was extracted from liver, spleen, and cecum samples using the MolPure^®^ TRIeasy Plus Total RNA Kit following the manufacturer’s protocol. Quantitative PCR analysis was performed using primers listed in **Supplementary Table 1**, with β-actin serving as the internal reference gene for normalization. Relative mRNA expression levels were calculated employing the comparative threshold cycle (2^−ΔΔCT^) method.

#### 2.8.5 16S rRNA gene sequencing assay

16S rRNA gene sequencing was performed as previously described (Chen et al., 2024). To investigate the impact of *S*. Typhimurium *tldi1* deletion on bacterial colonization and intestinal microbiota composition, cecal contents were collected from challenged mice on 5 dpi (Section 2.7.2), Samples were snap-frozen immediately in liquid nitrogen. Samples were submitted to Weike Meng Technology Group Co., Ltd (Shenzhen, China) for 16S rRNA gene sequencing and metagenomic analysis, which were conducted on the Illumina platform.

### 2.9 Data processing and statistical analysis

Statistical analyses were conducted using BM SPSS software (SPSS Inc., Armonk, NY, USA), with intergroup differences assessed by Student’s *t*-test or one-way ANOVA as appropriate. Values of P ≤ 0.05 or 0.01 were statistically significant or highly significant, with values presented as mean ± SD. All figures were generated using GraphPad Prism software (GraphPad Software Inc., San Diego, CA, USA) version 5.0.3.

## 3 Result

### 3.1 Bioinformatics analysis of Tldi1

#### 3.1.1 Physicochemical properties and hydrophilicity/hydrophobicity

**Figure 1:**
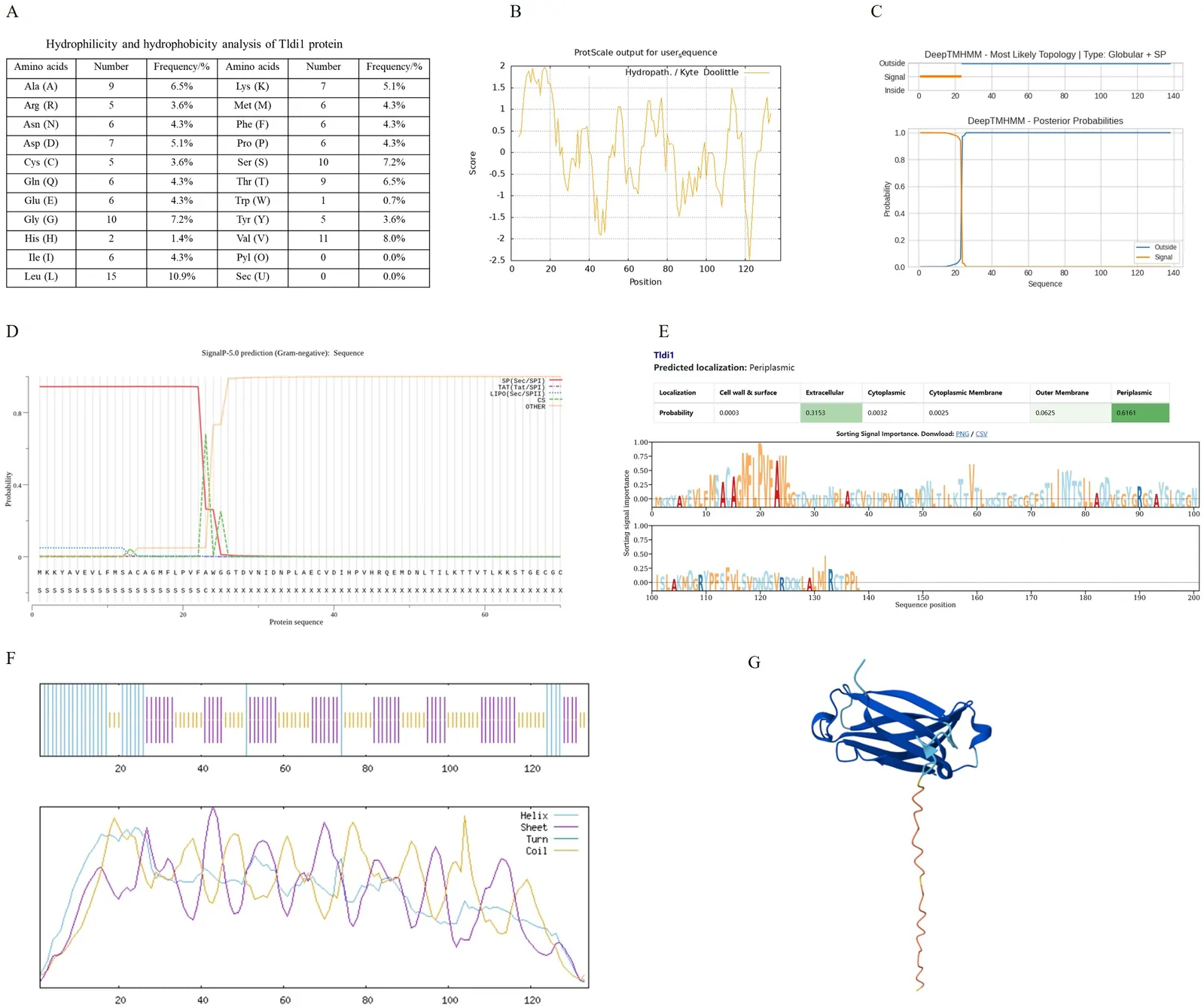
Bioinformatics analysis of Tldi1 protein. **(A)** Amino acid composition of Tldi1 protein; **(B)** Analysis of the hydrophilicity and hydrophobicity of Tldi1 protein; **(C)** Prediction of transmembrane region of Tldi1 protein; **(D)** Prediction of signal peptides of tldi1 protein; **(E)** Subcellular localization prediction of Tldi1 protein; **(F)** Prediction of secondary structure of Tldi1 protein; **(G)** Prediction of the tertiary structure of Tldi1 protein.

Bioinformatic analysis of the Tldi1 protein’s amino acid sequence predicted a total length of 138 residues (**Figure 1A**). Hydropathy profiling revealed maximum hydrophilicity at position 122 (score: -2.500) and strongest hydrophobicity at position 17 (score: 1.978). The comprehensive hydropathy profile demonstrated an overall hydrophilic character despite containing distinct hydrophobic regions (**Figure 1B**), suggesting potential membrane association or surface-exposed domains in the protein’s native structure.

#### 3.1.2 Analysis of transmembrane structures and signal peptides

Bioinformatic analysis of Tldi1 using TMHMM Server v.2.0 revealed a probable transmembrane domain spanning residues 5–27 (**Figure 1C**), suggesting membrane localization with the remaining C-terminal region (28–138) projecting extracellularly. Parallel SignalP-5.0 analysis identified a high-probability Sec-dependent signal peptide (score: 0.9455) with predicted cleavage between positions 23-24, while effectively excluding TAT pathway (score: 0.0019), lipoprotein (score: 0.0479), or non-secretory (score: 0.0047) alternatives (**Figure 1D**). These comprehensive predictions establish Tldi1 as a likely single-pass transmembrane protein featuring a classical N-terminal signal peptide directing Sec-mediated translocation.

#### 3.1.3 Prediction of protein subcellular localization, secondary structure and tertiary structure

The amino acid sequence of Tldi1 protein was submitted to the online tool DeepLocPro-1.0 for subcellular localization analysis. As shown in **Figure 1E**, subcellular localization analysis suggests that Tldi1 resides in the periplasm. The secondary structure prediction of Tldi1 is presented in **Figure 1F**. The protein structure consists of 39.55% random coils, 38.06% extended strands and 22.39% α-helices, with no β-turns detected. The amino acid sequence of Tldi1 was imported into AlphaFold for tertiary structure prediction, and the predicted structure is displayed in **Figure 1G**. The predicted pTM score of 0.74 by AlphaFold-Multimer indicates that the global folding quality of the Tldi1 protein is high. In AlphaFold-Multimer, pLDDT scores are categorized into four confidence levels: dark blue (>90, high confidence), light blue (70–90, moderate confidence), yellow (50–70, low confidence), and orange (0–50, very low confidence). According to the predicted structural model, Tldi1 exhibits high overall prediction accuracy. The structure of the Tldi1 protein predicted by AlphaFold-Multimer reveals that its core region consists of a high-confidence β-barrel fold (pTM = 0.74, core region pLDDT > 70), while a long flexible α-helix exists at the C-terminus (pLDDT 50–70), suggesting that this region may be an intrinsically disordered region (IDR) involved in dynamic regulation or membrane binding. The overall quality of the structural prediction is high, providing a reliable three-dimensional model basis for subsequent functional validation.

### 3.2 Construction of the SL1344Δ*tldi1* deletion strain

**Figure 2:**
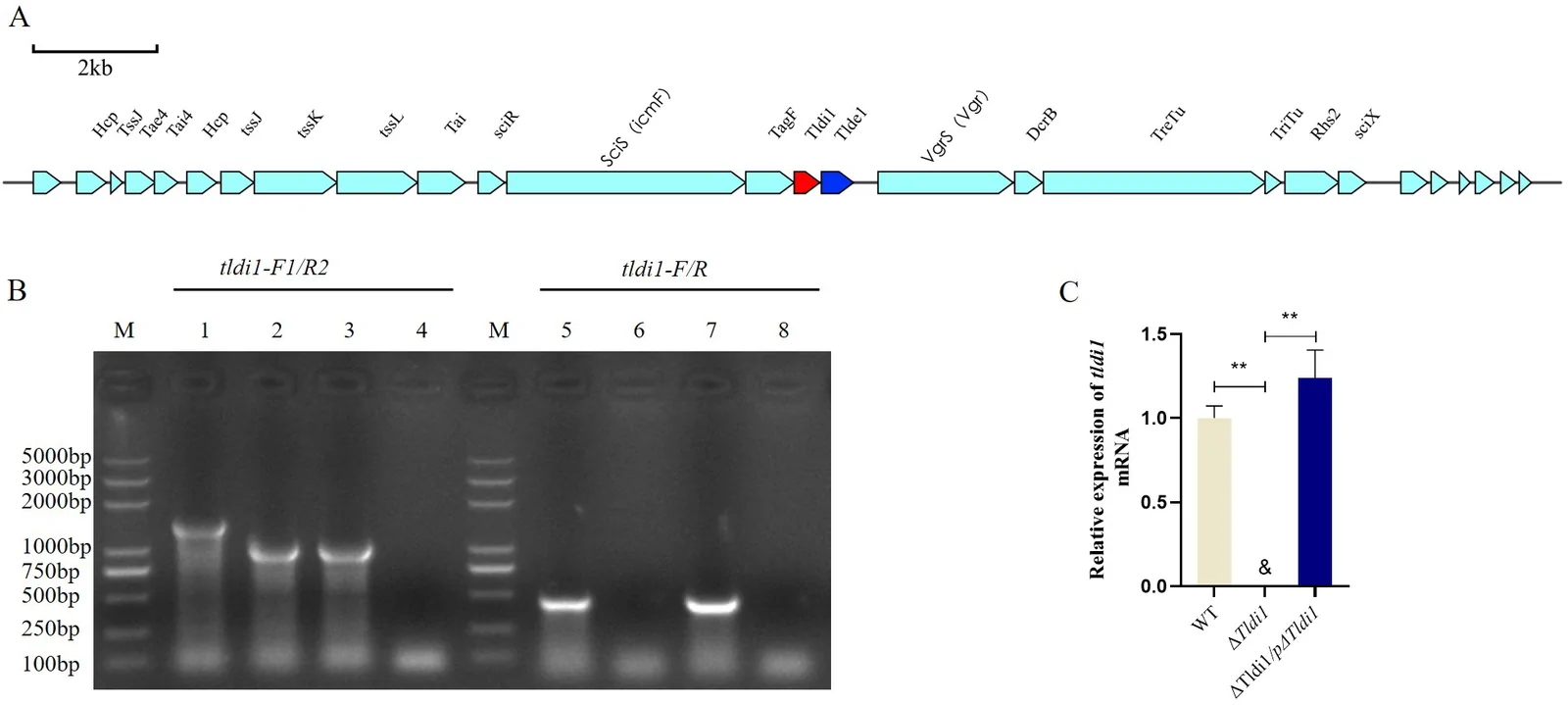
Construction of the *S.* Typhimurium *tldi1* deletion strain. **(A)** The location of the *tldi1* in the *S.* Typhimurium T6SS gene cluster; **(B)** The construction results of the *tldi1* knockout strain. M: DL2000PLUS DNA marker; 1, 5: WT; 2, 6: Δ*tldi1*; 3, 7: Δ*tldi1*/*pΔtldi1*; 4, 8: Negative control; **(C)** *tldi1* gene knockout was verified by qPCR. &: No data; statistical significance was determined using Student’s *t*-test, **P < 0.01.

The genomic location of the *tldi1* within the *S*. Typhimurium SL1344 cluster is illustrated in **Figure 2A**. Using the SL1344 genome as template, we generated a Δ*tldi1* deletion strain via homologous recombination and subsequently constructed complementation strain Δ*tldi1/pΔtldi1* by transforming Δ*tldi1* with a pBR322-*tldi1* recombinant plasmid. Successful generation of both Δ*tldi1* and the complementation strain was confirmed through PCR analysis (**Figure 2B**) and further validated by qPCR (**Figure 2C**). Primer pair *tldi1*-F1/R2 amplified a 1419 bp product containing the target fragment exclusively in wild-type SL1344, while Δ*tldi1* and its complementation strain produced only the homologous recombination flanking regions. Using *tldi1*-F/R primers, we detected a 419 bp target gene fragment in WT and complemented strains but not in Δ*tldi1*. Quantitative analysis of tldi1 mRNA expression confirmed the absence of *tldi1* transcripts in the deletion strain (**Figure 2C**), demonstrating successful construction of a functional *tldi1* knockout in *S*. Typhimurium.

### 3.3 Deletion of the *tldi1* gene alters the environmental tolerance of *S*. Typhimurium

**Figure 3:**
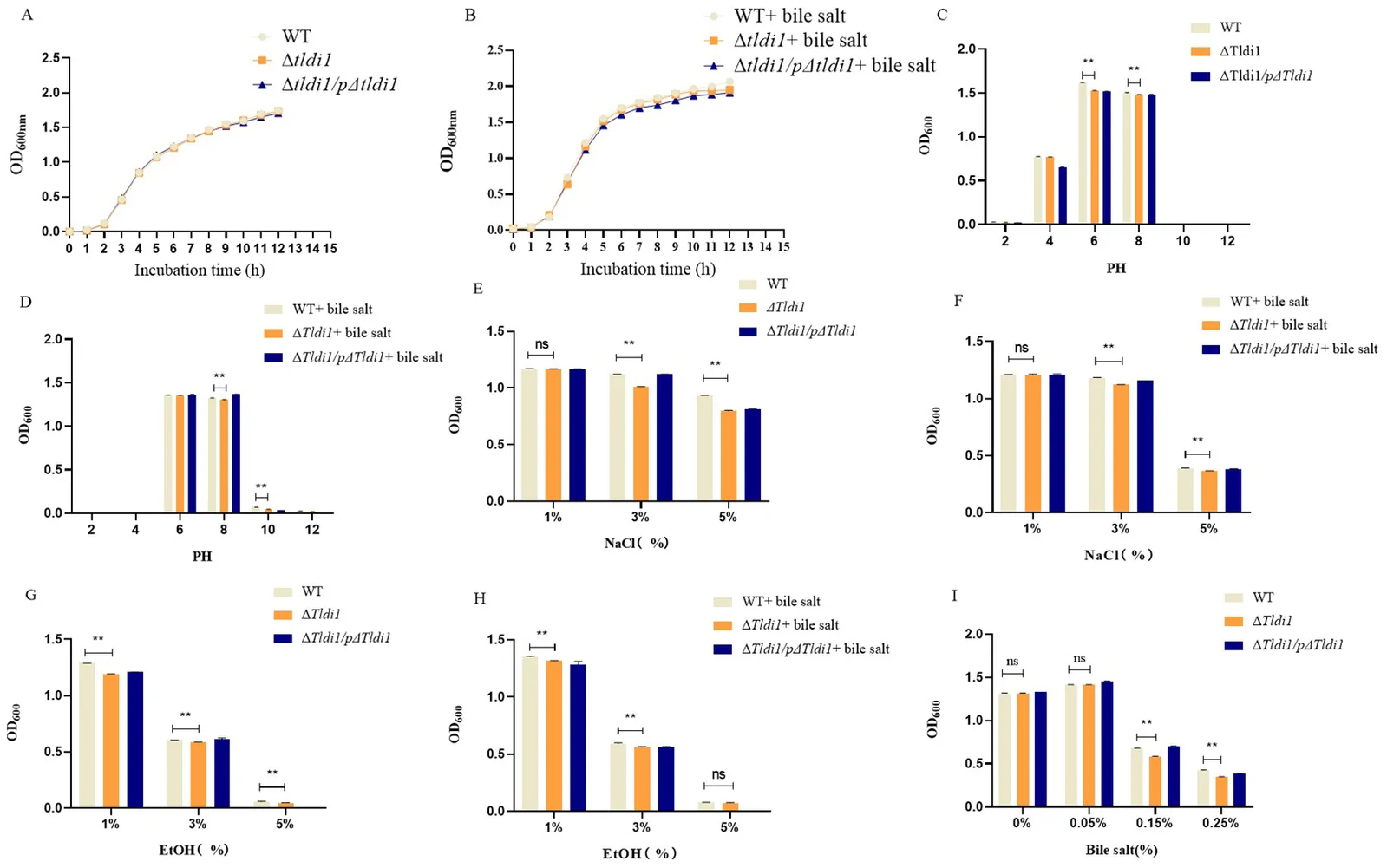
Environmental tolerance analysis of the *tldi1* gene knockout strain. **(A)** Growth curve of Δ*tldi1* in LB medium; **(B)** Growth curve of Δ*tldi1* in LB medium containing bile salts; **(C)** Acid-base tolerance test of Δ*tldi1* against *S.* Typhimurium in LB medium; **(D)** Acid-base tolerance test of Δ*tldi1* against *S.* Typhimurium in LB medium containing bile salts; **(E)** Salt stress tolerance test of Δ*tldi1* against *S.* Typhimurium in LB medium; **(F)** Salt stress tolerance test of Δ*tldi1* against *S.* Typhimurium in LB medium containing bile salts; **(G)** Ethanol stress tolerance test of Δ*tldi1* against *S.* Typhimurium in LB medium; **(H)** Ethanol stress tolerance test of Δ*tldi1* against *S.* Typhimurium in LB medium containing bile salts; **(I)** Bile salt stress tolerance test of Δ*tldi1* against *S.* Typhimurium in LB medium; Statistical significance determined by Student’s t-test, **P < 0.01, ns: *P* > 0.05.

To investigate the effect of *tldi1* gene deletion on the environmental stress resistance of *S*. Typhimurium. We plotted the growth curves of WT, Δ*tldi1*, and Δ*tldi1/pΔtldi1* in both normal LB medium and LB medium with bile salt added (**Figures 3A, B**). The results showed that the growth curves of WT, Δ*tldi1*, and Δ*tldi1/pΔtldi1* showed similar trends in normal environment and activated T6SS, indicating that the absence of *tldi1* gene did not alter their growth performance. When the growth environment of *S*. Typhimurium changes, it mainly includes acid stress, alkali stress (**Figures 3C, D**), salt stress (**Figures 3E, F**), hydrogen peroxide stress (**Figures 3G, H**), and pig bile salt stress (**Figure 3I**). The results showed that in ordinary LB medium, when pH=6 and 8, the growth of *S*. Typhimurium was significantly inhibited after the absence of *tldi1*gene (*P* < 0.01), and in LB medium with bile salt added, when pH=8 and 10, the growth of *S*. Typhimurium was significantly inhibited after the absence of *tldi1* gene (*P* < 0.01). With the increase of NaCl concentration, the growth of *S*. Typhimurium was significantly inhibited (*P* < 0.01) after the absence of *tldi1*. Under high concentration EtOH stress, the growth of *S*. Typhimurium was significantly inhibited (*P* < 0.01) after the absence of *tldi1*. Under bile salt stress, low concentrations of bile salt stress did not alter the growth of *S*. Typhimurium, while high concentrations of bile salt stress significantly inhibited the growth of *S*. Typhimurium without *tldi1*.

### 3.4 Changes in motility and biofilm formation of *S*. Typhimurium after knockout of the *tldi1* gene

**Figure 4:**
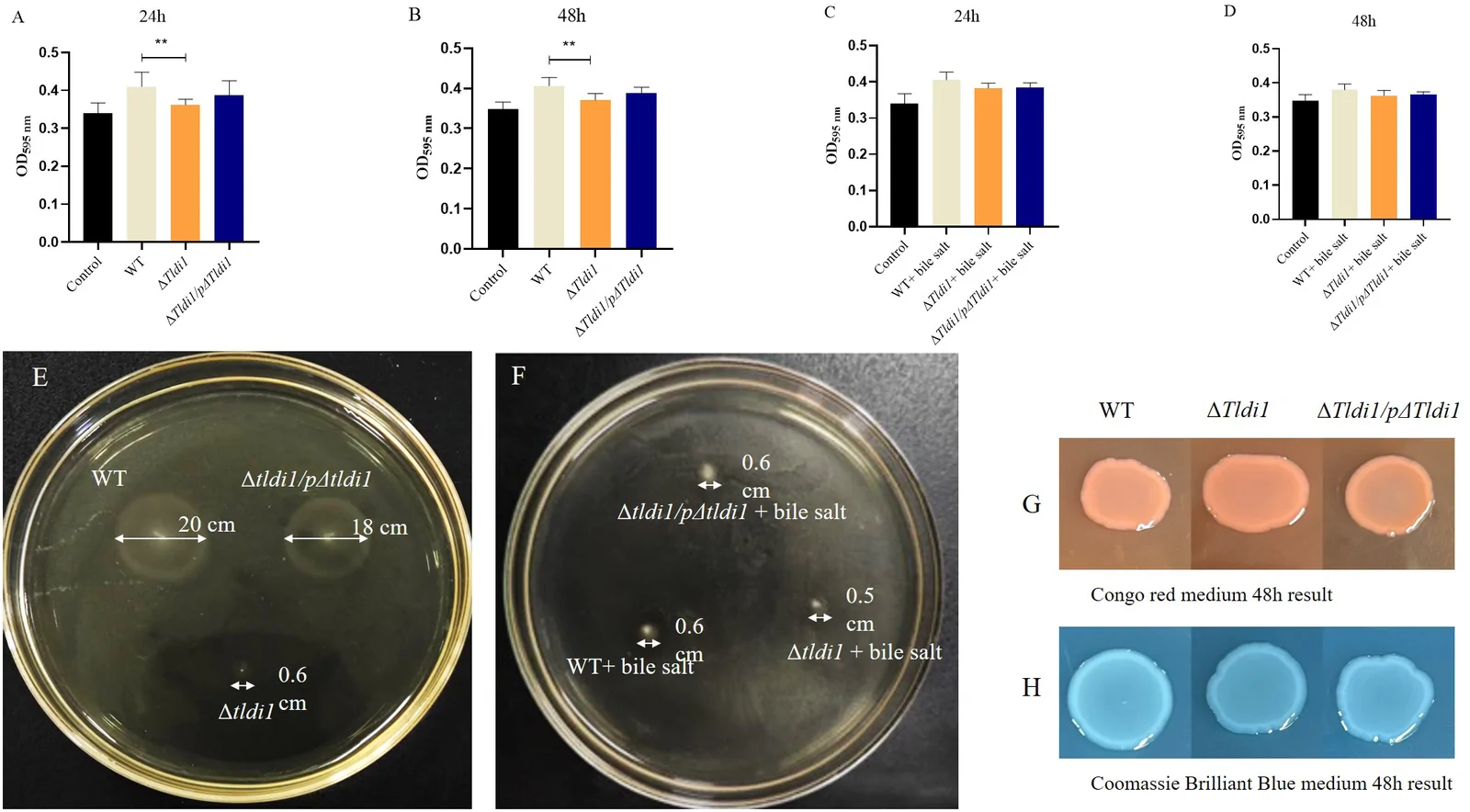
The effect of *tldi1* gene deletion on the biofilm formation and motility of *S.* Typhimurium. **(A, B)** Detection of the ability of *S*. Typhimurium to form biofilms after 24h and 48h of *tldi1* gene knockout; **(C, D)** Detection of the ability of *S*. Typhimurium to form biofilms in LB medium containing bile salts after 24h and 48h of *tldi1* gene knockout; **(E)** Detection of the motility of *S*. Typhimurium after *tldi1* gene knockout; **(F)** Detection of the motility of *S*. Typhimurium in LB medium containing bile salts after *tldi1* gene knockout; **(G)** Determination of curli pili in *tldi1* gene knockout strains; **(H)** Determination of extracellular proteins in *tldi1* gene knockout strains; statistical significance was determined using Student’s *t*-test, **P < 0.01.

Phenotypic characterization following *tldi1* knockout revealed significant alterations in biofilm formation and motility properties of *S*. Typhimurium SL1344. Quantitative biofilm assay demonstrated that compared with WT strains, after incubation for 24 h (*P* < 0.01) and 48 hours (*P* < 0.01) under standard LB conditions, the biofilm formation of Δ*tldi1* was significantly reduced compared with WT (**Figures 4A–D**), while supplementation with bile salts eliminated these differences. Motility assays revealed similar impairments. Compared with the WT strain, the Δ*tldi1* mutant exhibited significantly reduced swimming motility on soft agar plates, whereas no significant difference in motility was observed between the Δ*tldi1/p*Δ*tldi1* complemented strain and WT (**Figures 4E, F**). Interestingly, exposure to bile salts universally suppressed the motility of all tested strains. Neither Congo red binding assays nor Coomassie brilliant blue staining revealed differential extracellular matrix production between strains (**Figures 4G, H**) The absence of *tldi1* gene reduced the motility and biofilm formation ability of *S.* Typhimurium, as well as the ability of the strain to colonize and adapt to the environment; The extracellular matrix components remain unchanged, and bile salts have a global regulatory effect on exercise behavior, replacing genotype specific differences.

### 3.5 Differences in LD_50_ of *S*. Typhimurium after knockout of *tldi1* gene

**Figure 5:**
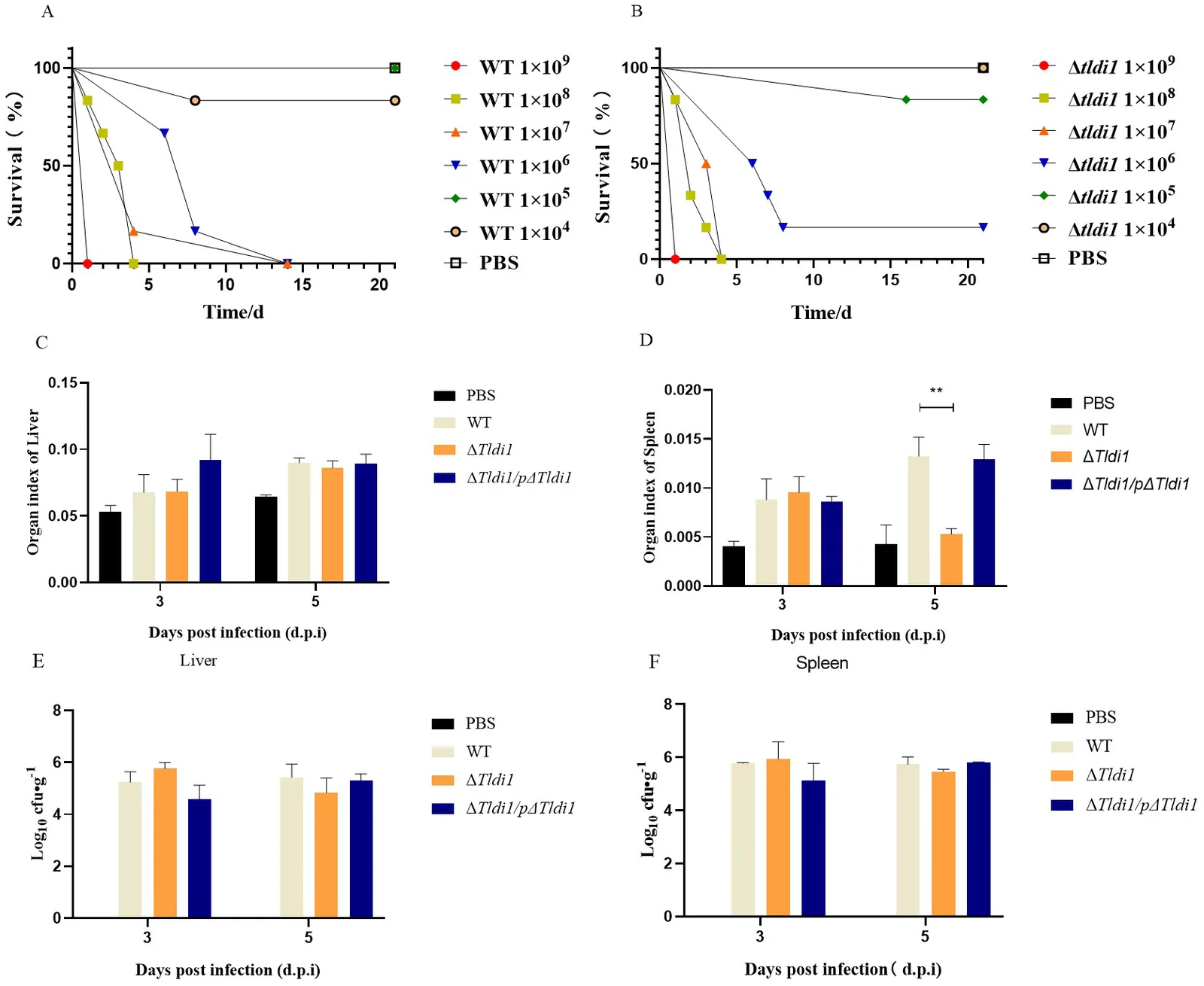
The effect of *tldi1* gene deletion on the virulence of *S.* Typhimurium in mice. **(A)** Survival rate of mice 21 days after infection with WT; **(B)** Survival rate of mice 21 days after infection with Δ*tldi1*; **(C)** Liver organ index of mice; **(D)** Spleen organ index of mice; **(E)** Bacterial load analysis in mouse liver; **(F)** Bacterial load analysis in mouse spleen; Statistical significance was determined using Student’s *t*-test, **P < 0.01.

A murine infection model was established to evaluate virulence attenuation following Tldi1 deletion in *S*. Typhimurium SL1344, with median LD_50_ determination revealing significantly reduced pathogenicity in the mutant strain. The LD_50_ value of the WT group was 1.8×10^5^ CFU, while that of the Δ*tldi1* group was 3.2×10^5^ CFU, with no statistically significant difference detected between them. However, the survival curves showed that at a challenge dose of 1×10^6^ CFU, the WT group caused 100% mortality in mice by 14 dpi, whereas the Δ*tldi1* group still had surviving mice until 21 dpi. The overall survival trends were clearly distinct, indicating that the virulence of the deletion mutant was attenuated (**Figures 5A, B**). These quantitative findings substantiate the critical role of Tldi1 in maintaining full virulence potential during systemic *S*. Typhimurium infection.

Following intraperitoneal inoculation with 1×10^5^ CFU/mL bacterial suspensions, we assessed tissue colonization patterns and pathological responses in infected mice. Comparative bacterial culture of liver and spleen homogenates on both XLT-4 selective media and ampicillin-supplemented LB plates revealed comparable colonization levels between Δ*tldi1* and WT strains at 3 dpi and 5 dpi (**Figures 5E, F**). Organ coefficient analysis (calculated as organ-to-body weight ratio) demonstrated divergent pathological responses: while liver indices remained unaffected, Δ*tldi1*-infected animals exhibited significantly reduced spleen coefficients relative to WT controls by 5 dpi (*P* < 0.01) (**Figures 5C, D**). These findings suggest that while *tldi1* gene deletion preserves bacterial colonization capacity in major organs, it specifically attenuates splenic pathological responses during systemic infection, potentially reflecting altered host-pathogen interactions mediated by this genetic locus.

### 3.6 The *tldi1* gene deletion reduces systemic inflammatory cytokine levels

**Figure 6:**
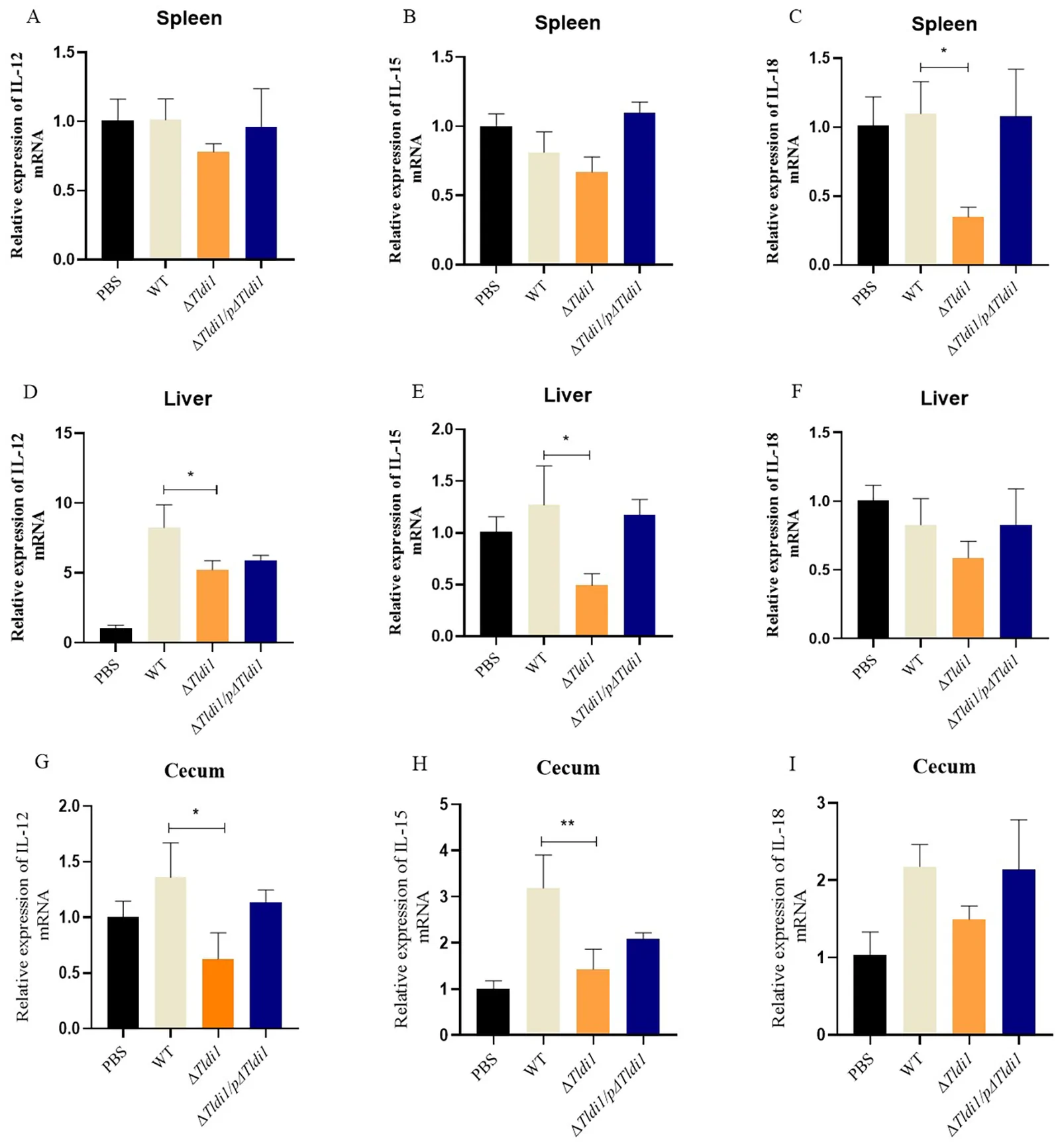
Expression of inflammatory factors in various organs after infection with Δ*tldi1* strain in mice. **(A)** Liver IL-12; **(B)** Liver IL-15; **(C)** Liver IL-18; **(D)** Spleen IL-12; **(E)** Spleen IL-15; **(F)** Spleen IL-18; **(G)** Cecum IL-12; **(H)** Cecum IL-15; **(I)** Liver IL-18; Statistical significance was determined using Student’s *t*-test, *P < 0.05, **P < 0.01.

To assess differential expression of inflammatory mediators following bacterial infection, we conducted RNA extraction from murine liver, spleen, and cecal tissues followed by reverse transcription quantitative PCR analysis. Comparative analysis revealed significantly elevated expression of immune-related cytokines in WT mice relative to Δ*tldi1* counterparts (*P* < 0.05). Specifically, splenic IL-18 expression demonstrated marked upregulation in WT mice (*P* < 0.05), while IL-12 and IL-15 levels remained statistically equivalent between groups (**Figures 6A–C**). Conversely, hepatic and cecal tissues exhibited profound WT-specific enhancement of both IL-12 and IL-15 expression (*P* < 0.01), though IL-18 levels showed no significant variation (**Figures 6D–I**).

### 3.7 Histopathological analysis of organ infection in *S*. Typhimurium-challenged mice following *tldi1* deficiency

**Figure 7:**
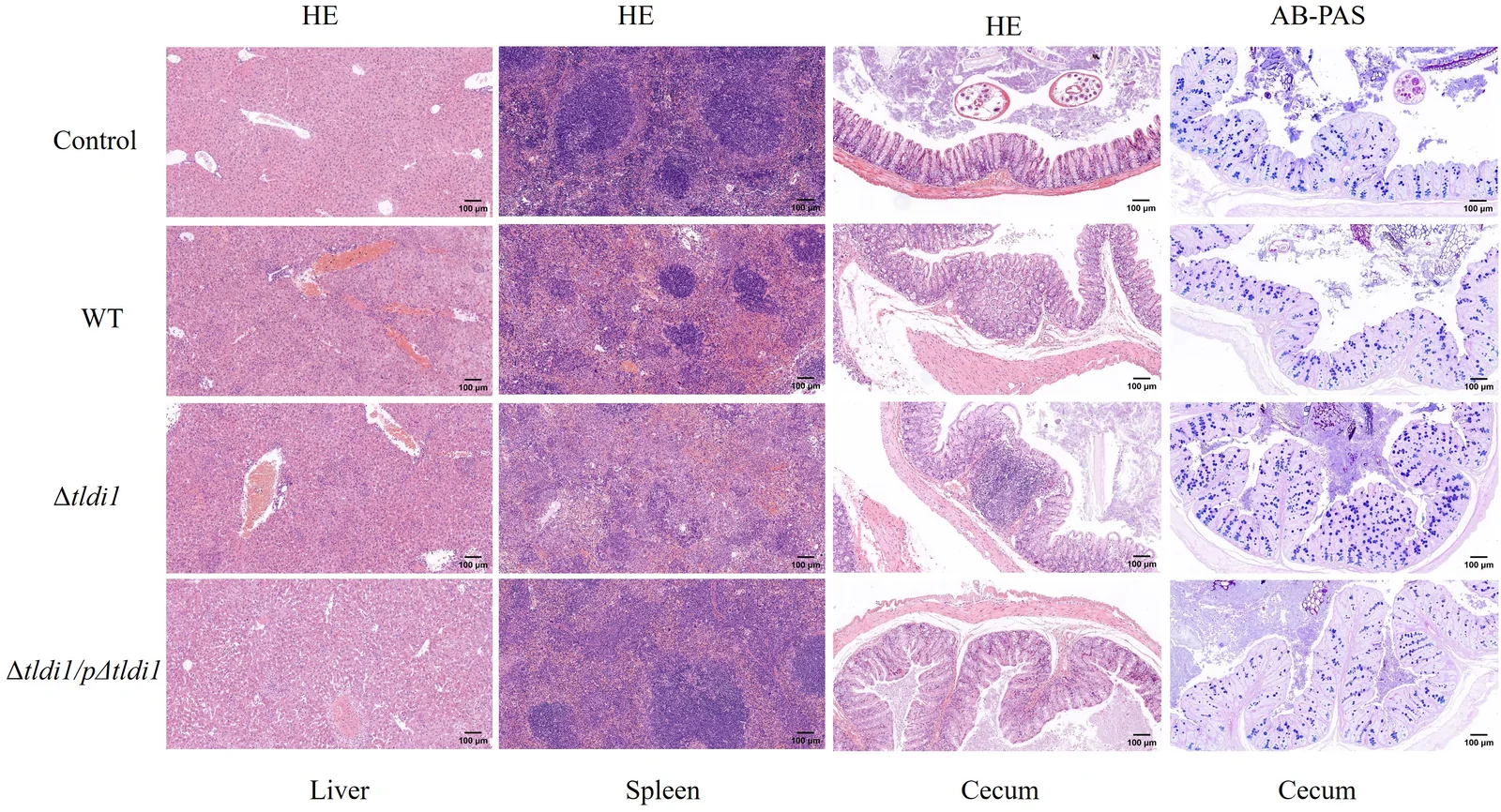
Pathohistological observations of various organs in mice infected with different strains.

Histopathological analysis of the liver, spleen, and cecum from intraperitoneally infected mice (**Figure 7**) demonstrated distinct differences across experimental groups. In the control (PBS) group, liver tissue exhibited normal architecture, characterized by polygonal hepatocytes arranged radially in orderly cords, intact central veins, and absence of inflammatory cell infiltration. By contrast, WT-infected mice displayed marked hepatic injury, including disorganized hepatocyte arrangement, indistinct cellular boundaries, central venous congestion, perivenular inflammatory cell infiltration, sinusoidal dilatation, and focal leukocyte aggregates. Liver pathology severity in Δ*tldi1/pΔtldi1*-complemented mice resembled that of the WT group. Splenic histology revealed preserved architecture in controls, with well-demarcated red/white pulp boundaries and distinct germinal centers. However, WT-infected mice exhibited erythrocyte accumulation within splenic sinuses and red pulp inflammation, while Δ*tldi1*-deficient mice showed attenuated lesions with maintained pulp boundaries and reduced sinusoidal erythrocyte retention. Cecal analysis identified severe mucosal disruption in WT animals, featuring villous disarray, submucosal leukocyte infiltration, muscularis-lamina propria separation, and goblet cell hyperplasia. These changes were significantly milder in Δ*tldi1* mice, which maintained near-normal villous integrity and goblet cell numbers comparable to PBS controls. The Δ*tldi1/pΔtldi1* group recapitulated WT-level cecal pathology, indicating genotype-dependent modulation of bacterial pathogenesis.

### 3.8 The gene *tldi1* deletion alters the gut microbiota in mice

**Figure 8:**
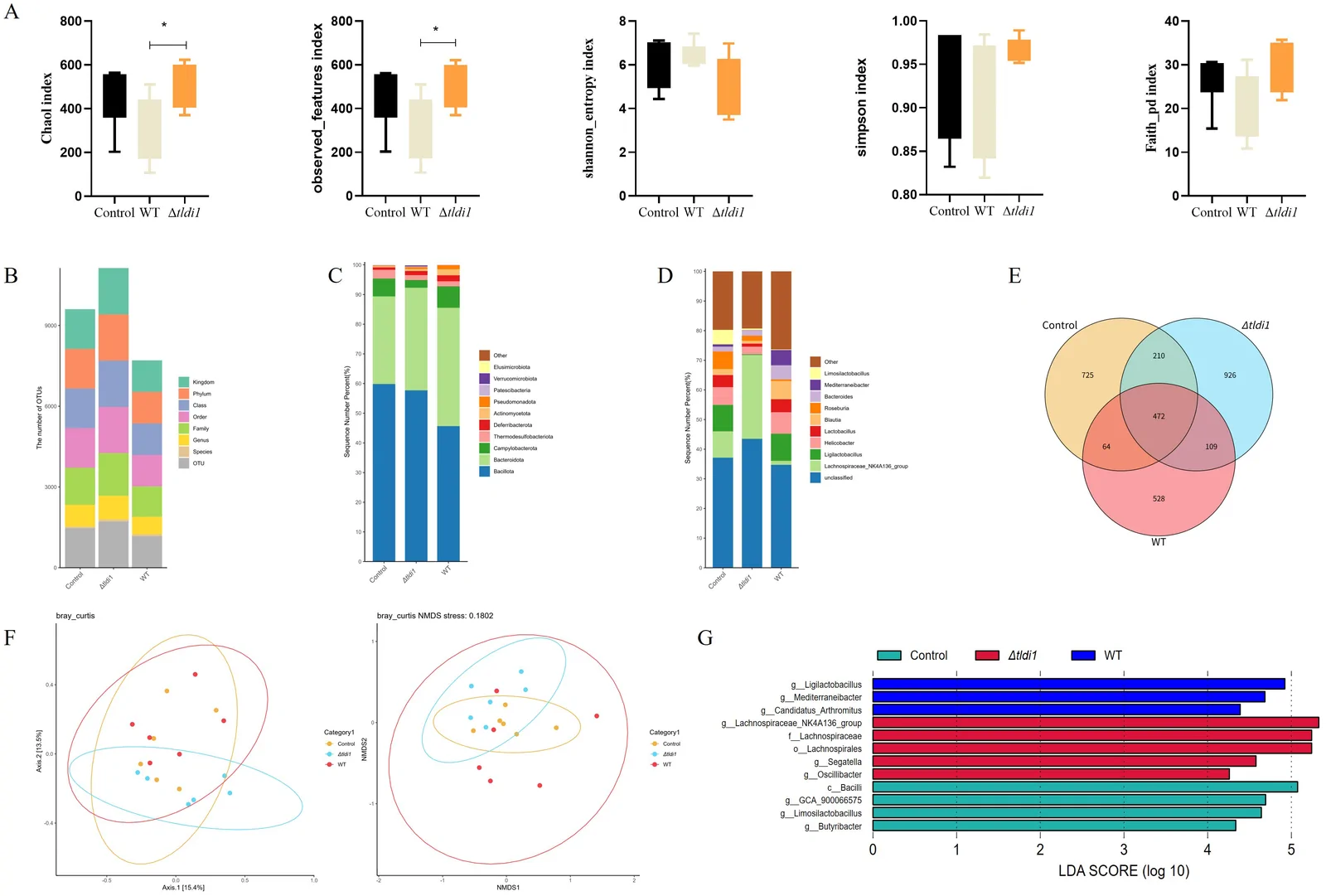
Δ*tldi1* affects gut microbiota balance. **(A)** Alpha Diversity Analysis: Chao1 index. Asses the actual number of species in the community, the larger the value, the higher the richness of the microbial community; Faith_pd index; observed_features index; shannon_entropy index and simpson index reveal the microbial diversity in the samples; the larger the shannon_entropy index, the greater the microbial diversity, whereas the simpson index showed the opposite trend; **(B)** Histogram of the distribution of intestinal flora in terms of domain, kingdom, phylum, class, order, family, genus and species; **(C)** Phylum level species relative abundance histogram; **(D)** Genus level species relative abundance histogram; **(E)** Venn diagram analysis of the relative abundance of intestinal flora; **(F)** Community composition of intestinal flora. NMDS analyzed the difference of microflora between different groups; **(G)** Differential species score plot in LEfSe analysis.

To investigate the potential impact of Tldi1 on gut microbiota composition, we performed high-throughput 16S rRNA sequencing on cecal contents collected from healthy mice inoculated with PBS (control), WT strain, or the Δ*tldi1* mutant strain. Comparative analysis revealed that *tldi1* gene deletion significantly altered gut microbial diversity in mice. Compared with the WT group, the Chao1 and observable d_features indices of the Δ*tldi1* group showed higher alpha diversity indices (*P* < 0.05) (**Figure 8A**). Principal coordinate analysis demonstrated distinct clustering patterns, indicating substantial differences in β-diversity between WT and Δ*tldi1* groups (**Figure 8F**).

Microbial composition analysis showed *Bacillota* and *Bacteroidota* as the predominant phyla. According to microbial community analysis, compared with the WT group, the Δ*tldi1* group displayed elevated bacterial abundance across all taxonomic levels including phylum, class, order, family and genus (**Figure 8B**). Notably, Δ*tldi1*-inoculated mice exhibited reduced abundance of *Bacteroidota* and *Campylobacterota* alongside increased *Bacillota* levels relative to WT controls (**Figure 8C**). At the genus level, we observed significant changes: *Ligilactobacillus*, *Helicobacter*, *Lactobacillus*, *Blautia*, *Bacteroides*, and *Mediterraneibacter* showed decreased abundance in Δ*tldi1* strain inoculated mice, while *Lachnospiraceae*_NK4A136_group and *Roseburia* populations expanded (**Figure 8D**). Venn diagram analysis identified 472 shared operational taxonomic units (OTUs) among all groups, with group-specific OTU counts of 725 (control), 528 (WT), and 926 (Δ*tldi1*), highlighting compositional differences (**Figure 8E**).

LEfSe (Linear Discriminant Analysis Effect Size) is a bioinformatics method used to identify differentially abundant taxa between groups with statistical significance (LDA score>2, p<0.05). LEfSe analysis further characterized group-specific microbial signatures: WT mice were enriched in *c_Bacilli*, *g_GCA_900066575, g_Limosilactobacillus*, and *g_Butyribacter*, whereas mice of Δ*tldi1* group showed distinctive enrichment of *g_Lachnospiraceae_NK4A136_group, f_Lachnospiraceae, 0_Lachnospirales, g_Segatella and g_Oscillibacter* (**Figure 8G**).

## 4 Discussion

*S.* Typhimurium is a typical pathogenic bacterium possessing multiple sets of T6SS. The T6SS is a contractile multiprotein complex that contributes to the environmental adaptability and virulence of a wide range of Gram-negative bacteria (Blondel et al., 2025). DUF-containing proteins are evolutionarily conserved across animals, plants, and microorganisms, participating in critical biological processes such as cellular regulation and metabolic homeostasis (Goodacre et al., 2013). Tldi1 (DUF2195) is an immunity protein of the T6SS. Nevertheless, its specific biological functions and underlying mechanisms during *S.* Typhimurium infection remain poorly elucidated. In this study, we constructed the *tldi1* knockout strain (Δ*tldi1*) and the complemented strain (Δ*tldi1/pΔtldi1*). By analyzing the phenotypic alterations of *S.* typhimurium, including environmental stress tolerance (acid-base pH, osmotic stress and ethanol resistance), biofilm formation and bacterial motility *in vitro*, we further explored the function of *tldi1* during bacterial infection using an *in vitro* infection model. Our results revealed that deletion of Tldi1 significantly impairs the stress adaptability of bacteria. The growth characteristics of *S.* Typhimurium showed no obvious changes after deletion of the *tldi1* gene. The growth of *S.* Typhimurium was restricted under acidic stress, alkaline stress, salt stress, bile salt stress and EtOH stress. With the increase in salt concentration, bile salt concentration and ethanol concentration, bacterial growth was progressively inhibited. Compared with the WT strain, the growth of the Δ*tldi1* strain was extremely significantly decreased (*P* < 0.01). A similar phenotype was also observed when the strains were cultured in LB medium supplemented with bile salts. In *Acinetobacter baumannii*, TagP is a PAAR protein located outside the core T6SS gene cluster. Previous studies have reported that deletion of the *tagP* gene significantly reduced bacterial survival under acidic conditions and high-salt stress, and simultaneously suppressed bacterial motility and biofilm formation capacity (Li et al., 2024a). In *Pseudomonas aeruginosa*, the hydrogen peroxide tolerance of the Δ*clpV3* mutant was significantly reduced (Lin et al., 2023). The T6SS4 deletion mutant of *Yersinia pseudotuberculosis* also exhibited high susceptibility to hydrogen peroxide (Zuo et al., 2023). In the motility assay, bacterial motility was markedly impaired following *tldi1* gene deletion. Upon supplementation of bile salts into the medium, growth of the WT, Δ*tldi1* and Δ*tldi1/p*Δ*tldi1* strains was all inhibited. Hcp is a crucial core structural protein of T6SS. Deletion of hcp has been reported to alter the motility of *S.* Typhimurium 14028s without affecting its growth characteristics (Wang et al., 2020). Evfg is an uncharacterized non-canonical protein in the T6SS of *E. coli*. Loss of the *evfg* gene attenuated bacterial motility, which may be attributed to reduced flagella synthesis (Zong et al., 2025). *Pseudomonas fluorescens* strain MFE01 secretes abundant Hcp1 and Hcp2. Deletion of hcp1 impairs bacterial motility, and Hcp1 can also enhance Hcp2-mediated bacterial killing capacity (Decoin et al., 2015). In the biofilm formation assay, the biofilm formation capacity of bacteria was decreased after Tldi1 depletion. Nevertheless, no significant difference in biofilm formation was observed upon bile salt addition. In *Vibrio alginolyticus*, in-frame deletion of the T6SS gene *VEPGS_0008* markedly reduced biofilm production (Liu et al., 2012). Similarly, clpV3 deletion in the H3-T6SS of *Pseudomonas aeruginosa* also significantly suppressed biofilm formation (Li et al., 2020). Functional deficiency of H3-T6SS and TepB reduced biofilm biomass and simultaneously weakened motility in *P. aeruginosa* PA14 (Yang et al., 2022). Previous studies have demonstrated that T6SS-3 of *Y. pseudotuberculosis* secretes Tce1, a novel Ca²^+^- and Mg²^+^-dependent DNase effector protein that degrades bacterial DNA (Song et al., 2021). Vpa1263, a T6SS effector protein from *Vibrio* parahaemolyticus, also possesses nuclease activity (Fridman et al., 2022). The effects of these proteins on extracellular DNA may collectively modulate bacterial biofilm formation.

To evaluate the role of Tldi1 in systemic pathogenicity, we first found no statistically significant difference in LD_50_ between the WT and mutant strains. Survival curve analysis demonstrated that deletion of the *tldi1* gene in *S.* Typhimurium slowed the lethal rate of the strain in mice, indicating a moderate trend of virulence attenuation. Subsequent analysis of organ indices demonstrated significant spleen atrophy (reduced organ index) in Δ*tldi1*-infected mice by 5 dpi, suggesting impaired immune function—confirmed histopathologically by milder lesions in liver, spleen, and cecum. Cytokine profiling further revealed immunological disruption: splenic IL-18 (key for Th1/NK cell activation) was significantly downregulated (*P* < 0.05), while hepatic and cecal IL-12/IL-15 (critical for Th1 differentiation and immune memory homeostasis) showed marked reductions (*P* < 0.01). All three are core cytokines central to Th1 immune responses. As an upstream immune initiator, IL-12 promotes the differentiation of naive T cells into Th1 cells and dominates the polarization of systemic immune responses. IL-18 synergizes strongly with IL-12 to robustly stimulate IFN-γ production and amplify subsequent immune reactions. IL-15 sustains the survival, proliferation and functional homeostasis of NK cells and effector T cells, extending the persistence of immune responses. With interrelated functions and hierarchical regulation, these three cytokines constitute an integrated Th1 immune regulatory axis. These data collectively indicate that Tldi1 deletion compromises (1) intracellular pathogen clearance, (2) immune memory maintenance, and (3) tissue-specific immune equilibrium.

Notably, despite reduced pathogenicity, liver/spleen bacterial burdens did not differ significantly between Δ*tldi1* and WT groups. This contrasts with reports where disruptions in Hcp2, PcgL, or SciS enhance *Salmonella* organ colonization (Mouslim et al., 2002; Parsons and Heffron, 2005; Wang et al., 2020), and ClpV deletion increases chick liver/spleen colonization (Chen et al., 2025). Conversely, analogous T6SS defects (e.g., TssS/TkeA in *Y. pseudotuberculosis*) reduce murine colonization and mortality (Song et al., 2025; Zhu et al., 2021), his is inconsistent with the results of this experiment. These paradoxical colonization patterns raise critical questions: Does Tldi1 primarily influence intestinal persistence rather than systemic spread? Is its virulence role interdependent with other secretion systems? Technical variables (sample size, measurement protocols, environmental conditions) may also contribute to these observed discrepancies. Future studies will address these unknowns through expanded sample sizes, optimized assays, and controlled experimental designs to clarify Tldi1’s mechanistic contributions to *Salmonella* pathogenesis.

Foodborne factors directly affect the structure of gut microbiota and are also the main pathway for *Salmonella* to enter the human body. Food contaminated with *Salmonella* is the main pathway for it to enter the human (Kogut and Fernandez Miyakawa, 2023). If the number of foodborne *Salmonella* exceeds the inhibitory capacity of gut microbiota, it will break through the barrier and cause infection. In Δ*tldi1* strain infected mice, gut microbiota α diversity increased, with higher species richness and more even species distribution within the intestinal microbiome. Campylobacter is a pathogenic bacterium in the gut that can disrupt the gut microbiota environment (Harrer et al., 2019), while Bacteroidetes and Firmicutes are dominant phyla in the gut that can maintain the homeostasis of the gut microbiota (Shin et al., 2024). In the mouse gut, relative abundance of *Campylobacterota* increased following *Salmonella* infection, leading to intestinal disruption. Conversely, relative abundance of *Campylobacterota* decreased in the guts of Δ*tldi1* strain infected mice, mitigating gut microbiota disruption. In WT mice, the relative abundance of Bacteroidetes decreased, indicating that the gut microbiota was affected by *Salmonella.* In the β-diversity analysis, the Δ*tldi1* group exhibited significant separation from the Control and WT groups, indicating substantial differences in microbial community structure among the three groups, each harboring distinct biomarkers. Collectively, these findings demonstrate that Δ*tldi1* exerts significant regulatory effects on both the diversity and structure of microbial communities.

This study focuses on Tldi1, a putative T6SS immunity protein in *S*. Typhimurium, investigating its functional role through the construction of a gene knockout strain (Δ*tldi1*). Key findings revealed that Tldi1 deletion significantly impaired bacterial motility, though its mechanistic link to flagellar regulation remains unexplored. Emerging evidence suggests three primary mechanisms through which T6SS may modulate motility: (1) Enzymatic activity: In *Vibrio cholerae*, the phospholipase effector Tle1^Vc^ enhances motility by upregulating flagellar genes (Liu et al., 2023). (2) Modulation of c-di-GMP signaling: This secondary messenger serves as a central regulator, integrating environmental cues to toggle bacteria between motile and sessile states (Paul et al., 2010; Srivastava et al., 2013; Yilmaz et al., 2020). Notably, in *Pseudomonas aeruginosa*, elevated c-di-GMP indirectly suppresses H1-T6SS while promoting flagellar motility (Zhou et al., 2022). (3) Signal transduction: For instance, PppA, a phosphatase in *Vibrio alginolyticus*, governs motility by dynamically regulating c-di-GMP levels (Sheng et al., 2013).

Based on these paradigms, we hypothesize that Tldi1 may (1) possess enzymatic activity capable of destabilizing membranes/cell walls, or (2) indirectly regulate flagellar function via c-di-GMP-mediated signaling. Future investigations will elucidate Tldi1’s molecular mechanisms through (1) enzymatic characterization, (2) transmission electron microscopy to assess flagellar morphogenesis (number, length, and assembly integrity), and (3) transcriptomic profiling of motility-associated genes and pathways. These studies aim to clarify whether Tldi1 modulates motility via structural disruption, transcriptional reprogramming, or post-translational signaling cascades. A limitation of this study is the lack of a Δ*tldi1/p*Δ*tldi1* double-deletion strain. However, previous studies have confirmed that Tldi1 specifically neutralizes Tlde1; thus, the phenotypes observed in the Δ*tldi1* strain are likely attributed to Tlde1-mediated self-intoxication. Future studies will incorporate double-deletion experiments to further validate this mechanism.

Our findings demonstrate that Tldi1 modulates environmental stress tolerance and motility in *S*. Typhimurium. Deletion of the *tldi1* gene showed no statistically significant effect on the overall virulence of *S.* Typhimurium, yet its virulence was attenuated in actual infection survival assays, and it induced significant alterations in the host’s multi-tissue immune responses. Although intestinal colonization remained largely unaffected in acute infection, Δ*tldi1* still induced tissue damage in the liver, spleen, and cecum. Collectively, these results suggest that Tldi1 does not directly govern intestinal colonization but may facilitate systemic dissemination and long-term infection persistence by regulating bacterial motility and environmental adaptability. Given its pivotal role in pathogenesis, Tldi1 represents a promising molecular target for mechanistic studies and attenuated vaccine development.

## Data Availability

The original contributions presented in the study are included in the article. The nucleotide sequence in this publication is available on GenBank accession number (GenBank: FQ312003.1; *Salmonella enterica* subsp. *enterica* serovar Typhimurium str. SL1344, c - Nucleotide - NCBI). 16S rRNA sequencing data are available in Genome Sequence Archive of China National Center for Bioinformation (PRJCA054435). Further inquiries may be directed to the corresponding author.
