# Correction: Tldi1 deficiency mediates Tlde1 self-toxic effect to regulate the adaptability of *Salmonella* Typhimurium infection and host immune response in mice

**DOI:** 10.3389/fcimb.2026.1963209

**Published:** 2026-08-25

**Authors:** Junfeng Zhang, Chang Liu, Songbiao Chen, Rongxian Guo, Yanyan Jia, Jing Li, Chengshui Liao, Ke Ding, Lei He, Ke Shang

**Affiliations:** 1 College of Medical Technology and Engineering, Henan University of Science and Technology, Luoyang, China; 2 College of Aimal Science and Technology/Laboratory of Functional Microbiology and Animal Health, Henan University of Science and Technology, Luoyang, China; 3 College of Animal Science and Technology, Key Laboratory of Live Carrier Biomaterials and Animal Disease Prevention and Control, Luoyang, China; 4 College of Animal Science and Technology, Henan Institute of Science and Technology, Xinxiang, China

**Keywords:** environmental stress adaptation, host-pathogen interactions, *Salmonella* Typhimurium, T6SS, Tldi1

Author “Yanyan Jia, Chengshui Liao” was erroneously included as corresponding author. The correct corresponding authors are “Lei He and Ke Shang”.

The original version of this article has been updated.

