# Supplementary material for: Tldi1 deficiency mediates Tlde1 self-toxic effect to regulate the adaptability of Salmonella Typhimurium infection and host immune response in mice
Source: Front Cell Infect Microbiol. 2026 Aug 3;16:1902089. doi: 10.3389/fcimb.2026.1902089 (PMC13478106; doi:10.3389/fcimb.2026.1902089)
Supplement: Supplementary file 1 [file Table1.docx]

Supplementary Material

# 1 Supplementary Tables

**Table S1**. The primer sequences used to generate and confirm SL1344Δ*tldi1* mutant strains

| Prime | Sequence（5'-3'） | Source |
| --- | --- | --- |
| *tldi1*-Up-F1 | GCTTATCGATACCGTCGACCCTCG ATGACCGGAGTGCTGATGGC | This study |
| *tldi1*-Up-R1 | TTCTGTGTACAGCATCTGACCTCTATTTTTTTC |  |
| *tldi1*-Down-F2 | CAGATGCTGTACACAGAATAGTCAGGGAGAAGA |  |
| *tldi1*-Down-R2 | CACTAGTGACGCGTACTCGATAAC CGTAAATGTCTTTTCACCA |  |
| *tldi1*-H-F | CCACACCCGTCCTGTGGATCCGTGAAAAAGTACGCTGTAGAGGTTCT |  |
| *tldi1*-H-R | TGCGTCCGGCGTAGAGGATCCTTACAGCGGTGGTGTACAACGT |  |
| *tldi1*-F | CTGGGGCGGAACAGATGTAA |  |
| *tldi1*-R | TCAACATCCTGCGCCAGTAA |  |
| invA-F | CAGGATACCTATAGTGCTGC |  |
| invA-R | CGCACCGTCAAAGGAACCGT |  |
| IL-12-F | TGGTTTGCCATCGTTTTGCTG | (Li et al., 2024) |
| IL-12-R | ACAGGTGAGGTTCACTGTTTCT |  |
| IL-15-F | ATGTTCATCAACACGTCCTGACT |  |
| IL-15-R | GCAGCAGGTGGAGGTACCTTAA |  |
| IL-18-F | AAATGGAGACCTGGAATCAGAC | This study |
| IL-18-R | TTTGTCAACGAAGAGAACTTGG |  |
| β-action-F | CCACGAAACTACCTTCAACTCC |  |
| β-action-R | GTGATCTCCTTCTGCATCCTGT |  |

**Reference:**

Li, Z., Qian, W., Zhang, Y., Liao, C., Chen, J., Ding, K., Yu, Q., Jia, Y., He, L., 2024. Enhanced Oncolytic Potential of Engineered Newcastle Disease Virus Lasota Strain through Modification of Its F Protein Cleavage Site. Microorganisms 12.

.

Table S2. Bacterial strains and plasmids used in this study.

| Strain or plasmid | Description | source |
| --- | --- | --- |
| SL1344 | *Salmonella enterica* *serovar* Enteritidis 1,4,5,12:i:1,2 | Our laboratory |
| SM10 | Constructing a vector for gene complementation strains; Amp^R^ | Our laboratory |
| Δ*tldi1* | *Salmonella enterica serovar* Enteritidis1,4,5,12:i:1,2Δ*tldi1* | This study |
| pDM4 | Suicide plasmid; Cm^R^ | Our laboratory |
| pBR322 | Constructing cloning vectors for ΔDUF2195/pΔDUF2195; Amp^R^ | Our laboratory |

Table S3. Bioinformatics software and web sites

| Software | web sites | Function prediction |
| --- | --- | --- |
| Protparam | https://web.expasy.org/protparam/ | Physicochemical properties of protein |
| ProtScale | https://web.expasy.org/protscale/ | Hydrophilicity/Hydrophobicity Analysis |
| TMHMM 2.0 | https://services.healthtech.dtu.dk/services/DeepTMHMM-1.0/ | Transmembrane Domain Prediction |
| SignalP5.0 | https://services.healthtech.dtu.dk/services/SignalP-6.0/ | Prediction of Signal Peptides |
| DeepLocPro-1.0 | https://services.healthtech.dtu.dk/services/DeepLocPro-1.0/ | Prediction of protein subcellular localization |
| SOPMA | https://npsa.lyon.inserm.fr/cgi | Prediction of protein secondary structure |
| AlphaFold | https://alphafoldserver.com/ | Protein structure prediction |
